# Teaching Engineering Ethics to PhD Students: A Berkeley–Delft Initiative: Commentary on “Ethics Across the Curriculum: Prospects for Broader (and Deeper) Teaching and Learning in Research and Engineering Ethics”

**DOI:** 10.1007/s11948-016-9809-7

**Published:** 2016-07-13

**Authors:** Behnam Taebi, William E. Kastenberg

**Affiliations:** 1 Department of Philosophy, Technology, Policy and Management, Delft University of Technology, PO 5015, 2600 GA Delft, The Netherlands; 2 Belfer Center for Science and International Affairs, Kennedy School of Government, Harvard University, 79 John F. Kennedy Street, Cambridge, MA 02138 USA; 3 University of California, Berkeley, Berkeley, CA 94720 USA

**Keywords:** Teaching ethics, Engineering ethics, Academic challenges, Institutional challenges

## Abstract

A joint effort by the University of California at Berkeley and Delft University of Technology to develop a graduate engineering ethics course for PhD students encountered two types of challenges: academic and institutional. Academically, long-term collaborative research efforts between engineering and philosophy faculty members might be needed before successful engineering ethics courses can be initiated; the teaching of ethics to engineering graduate students and collaborative research need to go hand-in-hand. Institutionally, both bottom-up approaches at the level of the faculty and as a joint research and teaching effort, and top-down approaches that include recognition by a University’s administration and the top level of education management, are needed for successful and sustainable efforts to teach engineering ethics.

## Introduction

Bringing ethics to the core of engineering curricula has received increasing attention over the last several decades. While systematic approaches to teaching engineering ethics were first developed in the U.S., they have expanded to include Europe and Australia since the 1990s (Zandvoort et al. 2000). An earlier special issue of *Science and Engineering Ethics* presented various examples of teaching social responsibility to science and engineering students in Europe (Bird et al. 2013). Papers included in the special issue of which this article is a part report on recent initiatives in various parts of the world, such as South-Africa, China, and more broadly in a globalized world.

In their paper “Ethics Across the Curriculum: Prospects for Broader (and Deeper) Teaching and Learning in Research and Engineering Ethics”, Carl Mitcham and Elaine Englehardt extensively review the history of *ethics across the curriculum* (EAC) in the U.S., of which engineering ethics is an “unacknowledged aspect” (Mitcham and Englehardt 2016). By comparatively reflecting on successes and failures at three American universities (Illinois Institute of Technology, Utah Valley University, and the Colorado School of Mines), they suggest that more “pedagogical research into what works in teaching and learning offers special opportunities”. The present paper reports on a joint effort by the University of California (UC) at Berkeley and Delft University of Technology (TU Delft) in the Netherlands to develop a graduate engineering ethics course for PhD students. Like Mitcham and Englehardt, we assert that graduate ethics education benefits most when it is a derivative of appropriate ethics research experience (see also Mitcham and Snieder 2014). The course was set up with three questions in mind: (1) What is the best way, pedagogically speaking, to teach engineering ethics to PhD students? (2) How can teaching and research in engineering ethics be efficiently combined? (3) How can one encourage collaborative (research) activities between PhD students from engineering and the humanities?

## History and Development of Engineering Ethics at TU Delft and UC Berkeley

At TU Delft there have been two decades of experience and goodwill among both the university administration and faculty members for teaching engineering ethics. By 1995, the Board of Directors had already decided that compulsory courses on ethical aspects of technology development and engineering must be introduced in all engineering departments at TU Delft (van de Poel et al. 2001). These developments are ongoing in that, in almost all engineering curricula at TU Delft, engineering ethics is included whether as an integral part of the final graduation thesis (of undergraduate [BSc] or Masters [MSc] students), as part of a course on societal aspects of engineering, or, in the most extensive form, as a stand-alone course on engineering ethics, either at the BSc. or the MSc. level. Since 2015, TU Delft has been further developing educational trajectories for teaching ethics; this concept is based on cumulative learning by including ethics in the existing engineering courses of each engineering curriculum.

There are several additional important initiatives and projects at TU Delft that are worth mentioning. Together with scholars from Eindhoven University of Technology and University of Twente, a web-based computer program was introduced for teaching engineering ethics (van der Burg and van de Poel 2005); a method was developed for systematically addressing moral problems in engineering called “the ethical cycle” (van de Poel and Royakkers 2006); and a leading textbook on engineering ethics, *Ethics, Technology and Engineering: An Introduction* was co-authored by Ibo van de Poel and Lambèr Royakkers (2011). TU Delft has also had experience with developing ethics programs that are co-taught by teachers from the department of philosophy and teachers from the engineering departments (Zandvoort et al. 2008). Finally, there has been increasing attention to offering courses on Value Sensitive Design and Responsible Innovation which essentially aim to combine engineering and ethics.

In response to ABET (formerly the Accreditation Board for Engineering and Technology) which requires an engineering ethics and social responsibility component for all accredited engineering and technology programs in the United States (ABET 2000), two new courses were developed at UC Berkeley: “Bioengineering 100” for bioengineering undergraduate majors, and “Engineering 124/125” for all engineering undergraduate majors. These courses were developed by senior faculty members and first taught in academic year 2002–2003, and they continue to be offered. In addition to foundational work in engineering ethics, “Bioengineering 100” focuses on specific issues related to patient rights, ethical conduct of research, and emerging issues in biotechnology. Recognizing that engineering students come from multi-cultural backgrounds and might eventually work for multi-national corporations, “Engineering 124/125” introduces students to a much broader view of ethics beyond Western philosophical moral theories (Hauser-Kastenberg et al. 2001).

The Minner Fellows Program for teaching engineering ethics and social responsibility was established in 2011 at Berkeley. The goal of the program is to enable selected faculty members to integrate engineering ethics and social responsibility into their capstone design and senior analysis courses. Each summer, up to six professors participate in an intensive short course taught in seminar fashion, and facilitated by faculty who are experienced in teaching ethics. To date, 18 faculty members have participated in the program.

In 2012, UC Berkeley was awarded a grant from the National Science Foundation for the project, “Making Ethics Explicit: Relocating Ethics to the Core of Engineering Education” (Sunderland et al. 2013). The project has two interrelated research and education components: (1) develop pedagogical approaches to engage with student’s emotions and existing value systems, and (2) explore how student-directed, problem-based learning can place ethics at the core of the curriculum (Sunderland 2014). The genesis of the project came from the observation that students already come to the university with an implicit set of values, assumptions and beliefs that lead to emotional responses to difficult ethical issues before engaging in moral reasoning (Sunderland 2014). UC Berkeley chose TU Delft as a collaborator because of its reputation regarding both the philosophy of emotion and the teaching of ethics at the graduate level.

## The First Edition of the Program at Berkeley: Fostering Interdisciplinary Collaborations

The first edition of the course “Global Perspectives: Engineering Ethics Across International and Academic Borders” was organized in Berkeley in August 2013. It was set up as a week-long intensive program with two goals: (1) to create research opportunities in the ethics of technology, and (2) to foster collaboration between different disciplines (mostly engineering and philosophy), and between PhD students from different universities. One key feature of the program was that it did not perceive students as objects of research, but instead as partners and co-inquirers in achieving the program’s goals. As such, the students were invited to contribute to engineering ethics scholarship by engaging in collaborative work while also addressing academic and institutional hurdles to such collaboration. This approach led to creating an open space for exploring opportunities while having a candid discussion about the difficulties associated with such opportunities (some of these difficulties are discussed in the remainder of this section).

An important innovation in organizing this program was that participants were invited to publicly reflect upon the workshop in commentaries that were published in the peer reviewed *Journal of Responsible Innovation*.^1^ There were also various collaborative initiatives started between PhD students during the program. To the best of our knowledge, only one of these initiatives led to a publication: “Just a cog in the machine? Interpreting nano-engineers’ forward-looking responsibility as a duty to collectivize” by three participants, Shannon Spruit, Gordon Hoople and David Rolfe (Spruit et al. 2016). Measuring the success of long-term academic exchange only in terms of publications is problematic and inappropriate because academic exchanges across disciplines can lead to creating awareness about ethical issues in engineering, and to broadening a professional’s network for future collaboration. Nevertheless, one could conclude that, to date, there was only one long-term substantial research opportunity created during the course.

The lack of more long-term collaborative research and a low level of participation from UC Berkeley were the key challenges of this program. These issues could be explained by two factors. First, interdisciplinary work in general (and more specifically ethics research) is often undervalued at engineering departments. This is partly a matter of perception, but this bias is sometimes reinforced by the difference in the impact factor of higher ranked disciplinary journals compared to often lower ranked (in terms of impact factor) interdisciplinary journals. This could lead to reservation among both senior scholars in engineering departments (whose movement up the academic ladder could be impeded) and junior scholars such as PhD students. Second, there was insufficient institutional recognition and support at the host institute: PhD students from UC Berkeley who participated in the program did not receive any form of credit for their participation. Participants from TU Delft did receive credit points from their graduate school that contributed to compulsory points required for graduation. In addition, transatlantic travel support was provided by the student’s home department at TU Delft.

One final element was the fairly high faculty/participant ratio: in total five faculty members were intensively involved in setting up and providing the program for twelve participants. While this was justified for a pilot program and for exploring collaborative possibilities between different disciplines, the group found this level of participation unsustainable without substantial funding dedicated to such programs, and perhaps even undesirable from the perspective of efficiency: the main goal of the program is to reach out to larger groups of junior scholars.

## The Second Edition of the Program in Delft: More Emphasis on Teaching

The second edition of this joint program was organized at TU Delft in July 2014. This edition had both a research and a teaching objective. The research objective was to explore new research areas in engineering ethics and to try to contribute to the scholarly literature. At the same time, the fruits of such a research endeavour could also help develop relevant cases and reading material for teaching engineering ethics to BSc. and MSc. students. Moreover, the course was meant to prepare PhD students for tutoring various engineering ethics courses at TU Delft. As mentioned earlier, the concept of teaching engineering ethics at TU Delft is based on co-teaching between the philosophy department and both senior and junior scholars from the engineering departments. Co-teaching by faculty from these two disciplines has three important benefits from the educational perspective. First, faculty members from engineering departments are often the ones who can best relate to and identify the ethical aspects of their own specific field of engineering. Second, on a related note, students often recognize that the involvement of scholars from engineering departments in teaching engineering ethics is an acknowledgement of the importance of the subject matter. Third, teaching engineering ethics as it has been set up at TU Delft requires substantial interaction with students regarding ethical issues in their field of engineering; often students attend interactive tutorials and write a short, single-authored or co-authored essay in order to explore one such issue in depth. This is a time-consuming teaching activity and a cascade model such as co-teaching can facilitate tutorials, while maintaining an intensive level of interaction with the students. An important benefit that co-teaching can have for PhD students who are involved as co-teachers is that they obtain valuable teaching experience that, in turn, is helpful for their future academic careers. In many engineering departments, PhD students do not have the opportunity to gain teaching experience during their PhD program.

This second version of the program was a collaboration between TU Delft and UC Berkeley, in the sense that it was taught by instructors from both institutions. Unfortunately, because of funding issues, it was not possible to have engineering students join from UC Berkeley. This program was successful in reaching out to PhD students at TU Delft without any extensive recruitment strategy. The maximum number of twenty participants (all from TU Delft) was even slightly exceeded.^2^ This was probably due to the institutional recognition of the course at TU Delft. When the course was set up in cooperation with UC Berkeley, a request was made to the board of the graduate school at TU Delft to assign credit points for it. PhD students at TU Delft are required to pass a minimum number of courses (most of which they take during the first year of their PhD program). Both editions of the program were identified as official courses of TU Delft’s graduate school. The second edition course at TU Delft was advertised through the official communication channels to PhD students. In the list of courses, it was labelled as ‘intellectual abilities’ of which students need to acquire a certain number of credit points. This made recruitment easy.

Unfortunately, the course was not successful in reaching the co-teaching goal. A number of graduate student participants did show interest in co-teaching, but they were unable to follow up after discussing the possibility with their thesis supervisors. This is presumably because of the time commitment required. Furthermore, the course did not manage to establish immediate scholarly activities in engineering ethics, although it might be too early to assess this because most participants were in the early stage of their PhD program. It should be noted that the department of philosophy at TU Delft has experience with PhD theses written as a result of collaboration between the philosophy department and the engineering departments (Bozdag 2015; Jacobs 2015; Schuurbiers 2010; Taebi 2010; van Gorp 2007) so there is a fair likelihood that program participants will reach out to the department of philosophy in later stages of their PhD program.

## Overcoming Institutional and Academic Challenges in Future Endeavours

The joint efforts between TU Delft and UC Berkeley to establish a graduate course in engineering ethics encountered two sets of challenges, one academic and the other institutional. With regard to academic challenges, the perception of interdisciplinary research (and more specifically engineering ethics) as second-rate academic work can be very problematic. A few years of groundwork might be needed in order to overcome this misguided view, as is evident from the paper by Mitcham and Englehardt (2016). In particular, there is a need for engineering departments to better understand and acknowledge the ethical issues in specific areas of engineering. This could require long-term contacts (and research efforts) between engineering and humanities (particularly ethics and philosophy) faculties. Such collaborations could increase goodwill among faculty members in departments of engineering, which will in turn have an indirect, positive impact on the PhD students’ recognition of such collaborative work as substantial, meaningful research. Hence, we recommend that the teaching of ethics to engineering students and collaborative research (between humanities and engineering faculties) go hand-in-hand.

Our second recommendation is aimed at institutional support and recognition of efforts in engineering ethics. It is important to note that both bottom-up and top-down approaches are needed. Engineering ethics (both its teaching and research) must be supported and organized at the level of faculty members and as a joint effort of engineering and humanities faculties. At the same time, recognition by a university’s administration and the top level of education management of engineering ethics activities could be helpful when organizing similar efforts. Indeed, this recognition could be in terms of facilitating (financially and otherwise) and formally integrating ethics courses into the official curriculum of the PhD program. Successful and sustainable efforts to teach engineering ethics require a balance between bottom-up and top-down approaches.

## Footnotes

1 These commentaries are “On Engineers Engaging Ethics through Dis-Location and Reconnection” (Bergen 2014); “Engineering Ethics in Every Decision” (Hoople 2014); “Technological Models: Epistemic and Responsible Perspectives” (Poznic 2014); “A Commentary on Engineering Ethics Education, or How to Bring about Change without Needing Scandals” (Robaey 2014); “No Field Is an Island: Why We Should Have Been Having an Interdisciplinary Discussion about Nano-Ethics All along” (Rolfe 2014); and “Responsible Innovation through Ethics Education: Educating to Change Research Practice” (Spruit 2014). The commentaries accompanied the paper “Making Ethics Explicit: Relocating Ethics to the Core of Engineering Education”, written by the instructors on the pedagogical findings of the workshop (Sunderland et al. 2014).

2 The Delft participants in the second edition were different than those in the 1st edition.
